# Cell Adhesion Molecules in Fibrotic Diseases

**DOI:** 10.3390/biomedicines11071995

**Published:** 2023-07-14

**Authors:** Qianjiang Hu, Komal Saleem, Jyotsana Pandey, Arzoo N. Charania, Yong Zhou, Chao He

**Affiliations:** 1 Division of Pulmonary, Allergy, Critical Care, and Sleep Medicine, Department of Medicine, University of Pittsburgh, Pittsburgh, PA 15213, USA; 2 Division of Pulmonary, Allergy, and Critical Care Medicine, Department of Medicine, University of Alabama at Birmingham, Birmingham, AL 35294, USA

**Keywords:** cell adhesion molecules, extracellular matrix, integrin, cadherin, pulmonary fibrosis, cirrhosis

## Abstract

Mechanisms underlying the pathogenesis of tissue fibrosis remain incompletely understood. Emerging evidence suggests that cell adhesion molecules (CAMs) are critical in fibrotic progression in many organs, including lung, kidney, skin, and liver. CAMs promote cell–cell and cell–extracellular matrix (ECM) interactions to maintain tissue architecture and normal function in homeostasis. However, dysregulated expression and function of CAMs can lead to chronic inflammation and tissue fibrosis. The major families of CAMs include integrins, cadherins, selectins, and immunoglobulins. Here, we review the role of the CAMs in fibrosis development across various organs with a focus on integrins and cadherins, and discuss their respective roles in the development of pulmonary fibrosis.

## 1. Introduction

Cell adhesion molecules (CAMs) are glycoproteins on the cell surface that are responsible for cell–cell and cell–extracellular matrix (ECM) interactions. CAMs help to maintain tissue architecture and promote normal cellular function in homeostasis. There are four main families of CAMs—integrins, cadherins, selectins, and immunoglobulins—each of which has its unique structure and function. Recent studies have found that CAMs play a critical role in tissue fibrosis across different organs. Understanding the role of CAMs will provide valuable insights into their contributions in tissue injury and repair processes and open avenues for potential therapeutic interventions.

## 2. Classes of Cell Adhesion Molecules

Cell adhesion molecules play a pivotal role in facilitating essential cell–cell and cell–matrix interactions. They are expressed on the surface of the majority of cell types, contributing to the regulation of cell junctions. Cell junctions include tight junction (TJ), adherens junction (AJ), gap junctions (GJ), and desmosomes, which are specialized structures that physically connect cells, providing mechanical strength to tissue. CAMs work in unison with other components of cell junctions to establish and maintain the overall tissue structure [1,2]. In addition to promoting cell–cell adhesion and maintaining tissue integrity, CAMs are also crucial for orchestrating cellular processes and regulating various physiological functions. Four main families of CAMs—integrins, cadherins, selectins, and immunoglobulins—have been identified, in which immunoglobulins’ functions are calcium-independent, whereas the functions of the other three are dependent on calcium.

### 2.1. Integrins

Integrins, a major class of transmembrane receptors, are essential in mediating cell–cell and ECM adhesion. Integrins are heterodimeric protein complexes consisting of α and β subunits. So far, 18 integrin α subunits and eight β subunits have been identified, and they can form 24 different integrins, each with specific binding properties and tissue distribution. Mammalian integrins are broadly classified as laminin-binding integrins (α1β1, α2β1, α3β1, α6β1, α7β1, and α6β4), collagen-binding integrins (α1β1, α2β1, α3β1, α10β1, and α11β1), leukocyte integrins (αLβ2, αMβ2, αXβ2, and αDβ2), and Arg-Gly-Asp (RGD)-recognizing integrins (α5β1, αvβ1, αvβ3, αvβ5, αvβ6, αvβ8, and αIIbβ3). Individual cells generally harbor multiple types of integrins on their surface, with each exhibiting distinct ligand specificities [3]. Serving as adhesion molecules, integrins anchor cells to the ECM to provide the mechanical support necessary for the regulation and maintenance of cell shape and tissue integrity. By binding to specific ECM proteins such as fibronectin, collagen, and laminin, integrins function as signaling receptors, activating intracellular pathways to regulate cell migration and differentiation, as well as tissue inflammation and repair [4]. Furthermore, integrins mediate cross-talk with other cell surface receptors, including growth factor receptors, cytokine receptors, and immune receptors, incorporating both extracellular and intracellular signals to modulate cellular responses [5,6]. Recent research has identified integrins as mechanosensors, which contribute to tissue remodeling by transducing mechanical forces from the ECM to the intracellular signaling pathways critical for fibrogenesis [7]. Intracellularly, integrins are associated with adopter proteins such as talin. Studies have shown that talin is required for integrin-mediated mechanosensing and mechanotransduction [8]. Talin is the principal protein that links integrins and intracellular F-actin, and integrin-mediated mechanotransduction is talin isoform-specific.

### 2.2. Cadherins

Cadherins are widely expressed in various cell types and are important in maintaining tissue integrity and enabling cellular interactions. Multiple types of cadherins have been identified, including epithelial (E-), neuronal (N-), placental (P-), vascular endothelial (VE-), retinal (R-), and mesenchymal (M-) cadherin. Structurally, cadherins possess three functional groups: (1) extracellular calcium-binding repeats that facilitate cell–cell adhesion; (2) a transmembrane domain for anchoring; and, (3) a cytoplasmic domain linking to the cytoskeleton [9]. Cadherins are further divided into two classes based on the presence (type I) or absence (type II) of a conserved His-Ala-Val (HAV) sequence in the extracellular domain. Type I cadherins include cadherin-1, cadherin-2, cadherin-3, and cadherin-4, and type II cadherins include cadherin-6, cadherin-7, cadherin-8, cadherin-9, cadherin-10, cadherin-11, cadherin-12, cadherin-18, cadherin-20, and cadherin-24. Cadherins are essential components of cell junctions, including adherens junctions and desmosomes, mediating calcium-dependent homophilic interactions between adjacent cells. These interactions promote the formation of strong adhesive contacts between cells, which uphold tissue cohesion and intracellular organization. The strength of these adhesive contacts is regulated by the concentration of calcium ions in the extracellular environment, which helps to ensure stable and robust cadherin-mediated adhesions [10,11,12]. Furthermore, cadherins participate in cellular signaling pathways, influencing cell, migration, differentiation, and function. Dysfunctional cadherins can disrupt tissue architecture and contribute to pathological conditions, including developmental disorders and tissue fibrosis [13,14].

### 2.3. Selectins

Selectins are a family of carbohydrate-binding proteins that mediate leukocyte rolling and adhesion to the endothelium during inflammation. Structurally, selectins are transmembrane glycoproteins characterized by five components: (1) an extracellular lectin domain, (2) an epidermal growth factor-like domain, (3) a variable number of short consensus repeats, (4) a transmembrane domain, and (5) a cytoplasmic tail. The lectin domain plays a crucial role in recognizing and binding to specific carbohydrate ligands on target cells, initiating cell–cell interactions. Three main types of selectins have been identified: P-selectin, E-selectin, and L-selectin. P-selectin is predominantly expressed on activated platelets and endothelial cells. E-selectin is induced on endothelial cells during inflammation. L-selectin is expressed on leukocytes. The interactions between selectins and their ligands, such as sialylated carbohydrates, mediate leukocyte rolling and subsequent adhesion, enabling leukocytes to migrate into tissue [15]. Selectins also participate in other physiological processes, such as hematopoiesis and tissue development. Dysregulation of selectin function has been associated with various diseases, including cancer metastasis and fibrosis [16,17].

### 2.4. Immunoglobulins

Immunoglobulin CAMs, also known as immunoglobulin superfamily (IgSF) CAMs, are involved in a wide range of cellular processes, including immune recognition, synaptic signaling, and axon guidance. Immunoglobulin-like cell adhesion molecules (IgCAMs) are the most diverse superfamily of CAMs. Unlike other CAMs, IgCAMs are calcium-independent and consist of extracellular domains with multiple immunoglobulin-like repeats that facilitate cell adhesion and binding interactions. The variable numbers of immunoglobulin-like repeats in IgCAMs contribute to their diverse adhesive properties and ligand binding capabilities. IgCAMs can mediate both homophilic and heterophilic interactions. Homophilic binding occurs when IgCAMs on adjacent cells bind to each other, while heterophilic binding involves the interaction of IgCAMs with other adhesion molecules or ligands [18]. The cytoplasmic region of IgCAMs contains signaling motifs that mediate bidirectional signaling across the plasma membrane. These motifs can interact with intracellular signaling molecules, leading to the activation of signaling pathways that modulate cellular function. Well-known members of IgCAMs include intercellular adhesion molecule (ICAM), neural cell adhesion molecule (NCAM), and vascular cell adhesion molecule (VCAM). ICAMs, including ICAM-1, ICAM-2, and ICAM-3, are involved in immune responses, facilitating leukocyte adhesion, migration, and interaction with endothelial cells. NCAM, expressed in the nervous system, plays crucial roles in neuronal development and synaptic plasticity. VCAM-1, expressed on endothelial cells, mediates leukocyte recruitment during inflammation.

## 3. Integrins in Pulmonary Fibrosis

### 3.1. αv Integrins

**Figure 1:**
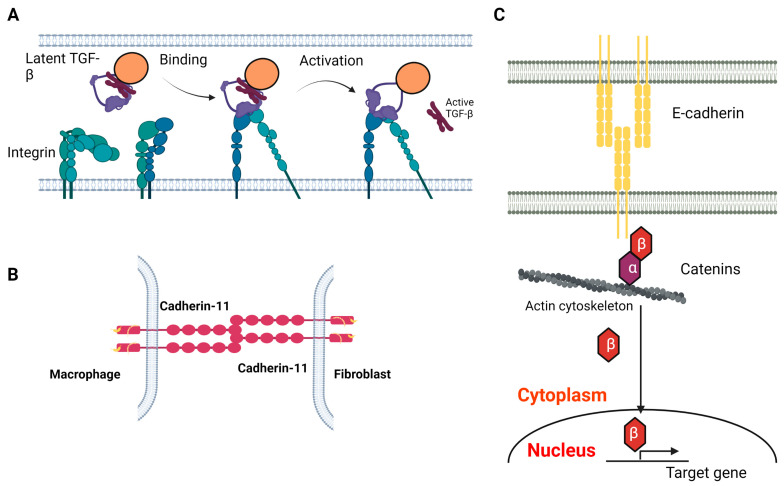
CAMs in fibrosis. (**A**) Integrin binds to latent TGF-b complex and releases active TGF-b. (**B**) The homophilic interaction between cadherin-11 on macrophages and fibroblasts. (**C**) E-cadherin–catenin complex. The figure was created with BioRender.com.

The αv integrins, including αvβ1, αvβ3, αvβ5, αvβ6 and αvβ8, which all harbor the RGD binding motif in their structure, are the most studied CAMs in tissue fibrosis. Mechanistically, αv integrins activate latent TGF-β via binding to the RGD sequence in the latency-associated peptide (LAP) of TGF-β (Figure 1A) [19,20]. Targeting αv integrin genetically or pharmaceutically has been shown to attenuate fibrosis in various organs, including lung and liver in vivo [21]. The most well-characterized αv integrin is αvβ6 integrin. In animals, β6 is minimally expressed in epithelial cells across several organs (lung, skin and kidney). Expression of β6 is augmented after lung injury induced by *Pseudomonas aeruginosa* infection, bleomycin exposure or radiation [19,22,23]. In humans, both type I and type II alveolar epithelial cells (AECs) have augmented αvβ6 expression in subjects with idiopathic pulmonary fibrosis (IPF) and pulmonary fibrosis associated with scleroderma compared with healthy subjects, while airway cells and macrophages have no αvβ6 expression in pulmonary fibrosis patients [24]. More importantly, increasing expression of αvβ6 in lungs correlates with mortality in patients with interstitial lung diseases (ILDs), suggesting it could serve as a prognostic marker [25]. Latent TGF-β activation requires an intact integrin αvβ6, as a truncated mutant lacking the β6 cytoplasmic domain fails to activate latent TGF-β [19]. In vivo, β6 global knockout mice are protected from developing pulmonary fibrosis after bleomycin exposure or radiation [19,23]. Similarly, β6 blockade by anti-αvβ6 antibodies via intraperitoneal injection protect mice from developing pulmonary fibrosis after bleomycin exposure or radiation, as well as in TGF-β overexpression transgenic mice [23,24,26]. Therapeutically targeting αvβ6 has been examined in several clinical trials. A trial using a subcutaneous injection of humanized anti-αvβ6 IgG1 monoclonal antibody (BG00011) was terminated in 2019 [27]. IPF subjects receiving BG00011 showed a worsening trend of FVC decline after 26 weeks, with increasing fibrotic changes on high-resolution CT and incidents of exacerbation despite reduction in TGF-β-mediated Smad activation [28]. It is known that loss of αvβ6 can potentiate lung inflammation and macrophage metalloelastase activation, which lead to emphysema in vivo [29]. It is unclear if the adverse events in subjects receiving αvβ6 antibody are associated with augmented lung inflammation. Recently, an inhaled selective small molecule targeting αvβ6, GSK3008348, has been developed [30]. GSK3008348 is an RGD-mimetic and highly selective for αvβ6 integrins compared with other αv integrins. In vitro, GSK3008348 induces internalization and lysosomal degradation of αvβ6 integrin in human bronchial epithelial cells. Ex vivo, GSK3008348 inhibits the canonical Smad pathway activation in precision-cut lung slides (PCLS) derived from IPF subjects. In vivo, inhaled GSK3008348 protects mice from developing pulmonary fibrosis after bleomycin exposure. A conjugate containing nintedanib and an αvβ6-recognizing small cyclopeptide has been developed, and has been shown to promote αvβ6 degradation and attenuate myofibroblast differentiation in vitro [31]. In addition to αvβ6, αvβ8 can also activate TGF-β. Unlike αvβ6, αvβ8 activates TGF-β independently of its cytoplasmic domain [32]. Instead, αvβ8 integrin activates TGF-β1 through regulating membrane-type 1 metalloproteinase (MT1-MMP) activity. The αv integrins are also expressed in fibroblasts, such as αvβ1, αvβ5, and αvβ3, while αvβ1 is expressed on pulmonary and hepatic fibroblasts and activates latent TGF-β. Targeting αvβ1 pharmaceutically is able to attenuate Smad pathway activation in fibroblasts and protect mice from developing pulmonary fibrosis after exposure to bleomycin [33]. A dual αvβ6 and αvβ1 inhibitor (PLN-74809) is currently in a phase 2 clinical trial [34]; αvβ5 can activate TGF-β in myofibroblasts and has been shown to contribute to persistent myofibroblast differentiation [35]. Mechanistically, integrin αvβ5 interacts with CD90, a cell surface glycoprotein also known as Thy-1 that contains the RGD sequence, in lung fibroblasts. Interruption of αvβ5 and CD90 interaction renders lung fibroblasts susceptible to TGF-β1 activation and myofibroblast differentiation. CD90 also interacts with αvβ3, a mechanosensitive integrin. In wild-type mice exposed to bleomycin, αvβ3 expression has a temporal correlation with fibrosis development and resolution. On day 14, there is increased αvβ3 expression in the lung. However, αvβ3 expression returns to baseline on day 42 when fibrosis has resolved. The binding of αvβ3 integrin to ECM enhances fibroblast activation via interactions between αvβ3 and CD90 [36]. In CD90 knockdown fibroblasts, increased amounts of αvβ3 localize to focal adhesions and maturation of soft ECM. In CD90 global knockout mice, αvβ3 expression is elevated, and mice develop progressive pulmonary fibrosis and have higher mortality after bleomycin exposure. In summary, αv integrins are expressed in epithelial cells and fibroblasts and have different functions in fibrogenesis, providing several targets for therapeutic development.

### 3.2. α6β1 Integrin

Increased ECM stiffness is the cardinal feature of various fibrotic diseases, including pulmonary fibrosis. Integrin α6 has been identified as a mechanosensitive integrin subunit that can be upregulated by stiff ECM and regulates myofibroblast invasion [37]. In healthy lungs, fibroblasts express little α6 integrin [38]. However, primary IPF lung myofibroblasts and myofibroblasts isolated from mice exposed to bleomycin express high levels of α6 integrin. Mechanistically, stiff matrix increases α6-integrin expression through activation of the Rho-associated protein kinase (ROCK)-dependent AP-1 transcription factor in fibroblasts, and α6β1 integrin subsequently mediates MMP-2-dependent proteolysis of collagen IV, leading to myofibroblast invasion through the basement membrane. In vivo, mice harboring a tamoxifen-induced fibroblast-specific deletion of α6 integrin are protected from developing pulmonary fibrosis after bleomycin exposure. Similarly, pharmacological inhibition of c-Fos/c-Jun within the AP-1 transcription factor protects mice against bleomycin-induced lung fibrosis. Targeting this mechanosensing α6β1 integrin offers a novel antifibrotic strategy against lung fibrosis.

## 4. Cadherins in Pulmonary Fibrosis

### 4.1. Cadherin-11

Cadherin-11 is a type II cadherin that plays a key role in tissue fibrosis. Cadherin-11 expression is increased in fibrotic tissue obtained from various organs including lung, skin, liver and kidney [39,40,41,42]. Cadherin-11 expression is increased in explanted lungs from patients with IPF, and in fibroblasts, AECs and alveolar macrophages within the fibrotic niche [14,40]. Similarly, cadherin-11 levels in lung homogenates were increased in mice exposed to bleomycin compared with mice treated with saline. Specifically, cadherin-11 expression is elevated in fibroblasts, AECs and alveolar macrophages from bleomycin-treated mice. Cadherin-11 contributes to lung fibrosis through multiple mechanisms. In vitro, TGF-β-treated AECs have increased expression of cadherin-11, N-cadherin and reduced expression of E-cadherin, consistent with transcriptomic changes observed in epithelial-to-mesenchymal transition (EMT), a process known to drive fibrosis development. Moreover, TGF-β treatment failed to induce EMT in epithelial cells deficient in cadherin-11, suggesting cadherin-11 is required for EMT [14]. Macrophages are the main source of TGF-β in pulmonary fibrosis [43,44]. Macrophages deficient in cadherin-11 produce less TGF-β compared with wild-type macrophages. Cadherin-11 is critical for the maintenance of the fibrotic niche (microenvironment), where TGF-β-producing macrophages and TGF-β-activating myofibroblasts form a feedforward loop via cadherin-11-mediated physical contact (Figure 1B). This cross-talk leads to persistent activation of myofibroblasts [40]. Silencing cadherin-11 in myofibroblasts abrogates interactions between myofibroblasts and macrophages. Similarly, inhibition of cadherin-11 using a neutralizing antibody can cause macrophage–myofibroblast separation, thereby destabilizing the fibrotic niche and attenuating myofibroblast activation. Recent studies have identified Forkhead box F1 (FOXF1) as a regulator of cadherin-11 in fibroblasts [45]. FOXF1 gene expression is reduced in fibroblasts isolated from human IPF lungs and lungs from mice exposed to bleomycin. Deletion of FOXF1 in myofibroblasts induces myofibroblast activation, migration and invasion in vitro and exacerbates bleomycin-induced pulmonary fibrosis in vivo. FOXF1 binds to the promoter region of the cadherin-11 gene and downregulates gene transcription, whereas N-cadherin transcription is upregulated. This FOXF1-regulated differentiated expression between N-cadherin and cadherin-11 leads to myofibroblast activation. In vivo, global cadherin-11 knockout mice were protected from developing pulmonary fibrosis after bleomycin exposure. Additionally, inhibiting cadherin-11 with neutralizing monoclonal antibodies through intraperitoneal injection attenuated established pulmonary fibrosis in bleomycin-treated mice [14]. It is known that the macrophage population promoting fibrosis is from bone marrow-derived monocytes, termed monocyte-derived macrophages (MDMs) [46]. Recently, cadherin-11 was found to be important for the differentiation of the bone marrow progenitors and the monocyte population. Specifically, cadherin-11 deficiency increases the number of common myeloid progenitors (CMPs), but reduces the number of granulocyte–monocyte progenitors (GMPs). Cadherin-11 deficiency led to attenuated production of TGF-β and reduced expression of *Arg1*, *Chil3* and *Retnla*, all profibrotic genes critical for fibrosis development [47].

### 4.2. E-Cadherin

E-cadherin, or cadherin-1, is predominantly expressed in epithelial cells and maintains the structural integrity of epithelial tissue by mediating homophilic interactions between adjacent cells. The extracellular domain of E-cadherin interacts with other E-cadherin molecules on adjacent cells, while the intracellular cytoplasmic tail of E-cadherin is associated with various catenins (α, β, and p120). The link between extracellular signals to the intracellular cytoskeleton provides mechanical stability to the tissue and modulates intracellular pathways such as Wnt, TGF-β and Hippo, all of which are implicated in pulmonary fibrosis [48,49,50,51]. Cigarette smoke extract or TGF-β treatment can suppress the expression of E-cadherin in epithelial cells in vitro, and E-cadherin expression is reduced in the end-stage fibrotic lungs of various etiologies [52,53,54,55,56]. As mentioned above, EMT is critical for fibrosis initiation and progression. During EMT, epithelial cells lose cell–cell connection and polarity due to reduced levels of E-cadherin. Cadherin cytoplasmic tails bind with β-catenin to form a cadherin–catenin complex (Figure 1C). During EMT, cells undergo cadherin switch from E-cadherin to N-cadherin, which leads to the release of active β-catenin and promotes cell proliferation [57]. The loss of cell–cell connection is mediated by E-cadherin and zonula occludens (ZO)-1 on the cell surface and is regulated by MUC1 glycosylation [58]. Mechanistically, studies have showed that TGF-β receptor, E-cadherin, and α3β1 integrin form a tri-molecular complex that regulates the Wnt/β-catenin pathway through β-catenin phosphorylation at the tyrosine 654 residue (Y654) in AECs. This results in β-catenin nuclear translocation, which activates the Smad2 pathway and initiates EMT [59]. Lung tissue from IPF subjects showed increased amounts of pY654-β-catenin in myofibroblasts. Moreover, mice harboring an epithelial-specific deletion of α3 integrin have attenuated fibrotic response with reduced numbers of myofibroblasts and collagen deposition, despite a normal inflammatory response after bleomycin exposure. This study also highlights that two CAMs, α3β1 integrin and E-cadherin, coordinate with each other to regulate fibrosis development.

## 5. Cams in Other Fibrotic Diseases

### 5.1. Liver Fibrosis

The liver possesses a remarkable ability to repair and regenerate itself after injury. However, if excessive stress is placed upon the liver through repeated injury, its capacity for repair can be overwhelmed, resulting in the replacement of damaged liver tissue with scar tissue. Liver fibrosis or cirrhosis is a prevalent pathological condition characterized by an accumulation of excessive ECM. Integrins play an important role in liver fibrosis by facilitating the activation of hepatic stellate cells (HSCs), which are the main ECM-producing cells in the liver. Upon activation, HSCs undergo a transformation from a quiescent phenotype to a myofibroblast-like phenotype, characterized by the expression of α-smooth muscle actin (α-SMA) and increased production of ECM components. Integrins such as αvβ3, αvβ5, and α5β1 are all involved in HSC activation and ECM production [60,61,62]. Integrins are the primary receptors that connect cells to ECM. The αvβ6 integrin plays a vital role in liver fibrosis, as it activates the latent TGF-β through its binding of the LAP. Moreover, integrins can regulate mechanical forces via the actin cytoskeleton, further promoting the release of active TGF-β from the latent complex [63]. In animal models, inhibition of integrin αvβ6 has been shown to reduce carbon tetrachloride (CCL4)-induced liver fibrosis [21,64]. Integrins also contribute to liver fibrosis via enhancing inflammation. Studies have suggested that CD4^+^ T lymphocytes with increased expression of CD11α (integrin alpha L) play a critical role in mediating proinflammatory responses in primary biliary cirrhosis (PBC) [65]. Furthermore, neutrophils exhibit upregulated expression of αMβ2 integrin after activation, which promotes chronic liver injury [66,67].

Cadherins are crucial for the maintenance of tissue structure and organization in the liver. Specifically, E-cadherin plays an essential role in forming the adherens junctions between HSCs and hepatocytes. Loss of these junctions can trigger the activation of HSCs, which leads to the progression of liver fibrosis [68]. On the contrary, hemophilic binding of E-cadherin down regulates the Hippo pathway and attenuates HSC activation. E-cadherin-neutralizing antibody treatment activates HSC in an organoid ex vivo. Animal studies using CCL4-induced liver fibrosis have demonstrated an increased expression of cadherin-11 in HSCs in CCL-4-treated mice, underscoring its role in HSC activation [41]. Additionally, decreased E-cadherin expression has been observed in cholangiocytes in primary sclerosing cholangitis (PSC), and liver-specific E-cadherin knockout mice have exhibited periportal inflammation and periductal fibrosis [69]. Selectins are commonly known to promote inflammation by recruiting leukocytes. In chronically ethanol-fed mice, E-selectin, but not other types of selectins, was found to be highly upregulated [70]. Platelet endothelial cell adhesion molecule 1 (PECAM-1), also known as CD31, is primarily expressed on leukocytes and endothelial cells. PECAM-1 mediates transendothelial migration of neutrophils and monocytes during inflammation. Studies have shown that PECAM-1-deficient mice exhibit a proinflammatory and exacerbated fibrogenesis phenotype in a high-fat-diet-induced nonalcoholic steatohepatitis (NASH) model [71]. In conclusion, CAMs play a critical role in the development and progression of liver fibrosis. Understanding the mechanisms by which CAM regulates HSC activation and migration may lead to the development of novel therapies for liver fibrosis/cirrhosis.

### 5.2. Dermal Fibrosis

Skin is the main barrier organ in humans. CAMs are critical in maintaining the structural integrity and function of skin. Integrin αvβ6 expression is strongly upregulated in the epidermis of chronic human wounds [72]. Scleroderma is an autoimmune disease characterized by progressive fibrosis that affects various parts of the body, including skin. EMT has been proposed as a key mechanism contributing to the development of scleroderma. E-cadherin expression is reduced during EMT, resulting in loss of cell–cell adhesion and tissue integrity, eventually leading to dermal fibrosis. Additionally, scleroderma skin fibroblasts have increased expression of αvβ3 and αvβ5 integrins [73,74,75]. These integrins activate the TGF-β signaling pathway, leading to dermal fibroblast differentiation into a myofibroblast phenotype that is capable of increasing collagen synthesis and deposition in the extracellular space. In a bleomycin-induced scleroderma animal model, L-selectin and ICAM-1 contribute to the development of skin fibrosis, whereas P-selectin, E-selectin, and P-selectin glycoprotein ligand 1 (PSGL-1) suppress the fibrosis process [76]. Junctional adhesion molecules (JAMs) are proteins of the immunoglobulin superfamily. Aberrant expression of JAMs was detected in skin of scleroderma patients. Moreover, circulating soluble JAM-C in the serum has been identified as a biomarker for worsening skin involvement in patients with scleroderma [77].

### 5.3. Kidney Fibrosis

In the kidney, CAMs are involved in the recruitment and activation of immune cells and the regulation of ECM remodeling. Integrins are the most extensively studied CAMs in kidney fibrosis. Integrin αvβ3 has been found to promote fibroblast proliferation and ECM production in diabetic nephropathy [78], while integrin αvβ5 regulates fibroblast migration and collagen deposition in renal interstitial fibrosis [79]. Fibronectin, a major component of the ECM, is responsible for matrix assembly. Integrins such as α5β1 bind to fibronectin to form excessive fibrils, causing tissue damage in the kidneys [80]. In physiological conditions, α3β1 integrin binds to E-cadherin in cell–cell adhesion to mediate cell–matrix interaction. Loss of E-cadherin in proximal tubular epithelial cells increases the expression of α3β1 integrin, which plays a critical role in TGF-β–induced profibrotic processes in the kidneys [81]. This study also highlights the coordinated efforts between E-cadherin and α3β1 integrin in fibrosis development, similar to the one observed in pulmonary fibrosis [59].

Other CAMs such as selectins, cadherins and immunoglobulins also play a role in kidney fibrosis. P-selectin promotes the migration and proliferation of myofibroblasts in the kidney by activating the TGF-β/Smad3 signaling pathway [82]. E-selectin is involved in the recruitment of monocytes to the kidney and the development of renal interstitial fibrosis in a mouse model of obstructive nephropathy [83]. ICAM-1 can promote the adhesion of immune cells to the surface of kidney cells, leading to the release of proinflammatory cytokines and the activation of fibroblasts, which are responsible for the excessive production of ECM [84]. Another important CAM in kidney fibrosis is VCAM-1. VCAM-1 promotes the adhesion and recruitment of immune cells and is upregulated in response to injury and inflammation [85]. Targeting CAMs may therefore represent a promising strategy for the prevention and treatment of kidney fibrosis due to their crucial roles in the inflammatory response and ECM remodeling.

## 6. Conclusions

Current research in the field of CAMs in fibrotic disorders has focused on several areas: (1) specific CAMs that are aberrantly expressed in different processes of fibrogenesis, including acute and chronic inflammation, initial and sustained fibroblast activation, EMT, and ECM remodeling; (2) the signaling pathway(s) and network(s) through which CAMs regulate fibrosis (several signaling pathways link with CAMs, including the Wnt/β-catenin signaling pathway and TGF-β signaling pathways, which are known to contribute to fibrosis progression); and (3) CAM-targeted therapies for various fibrotic disorders, including pulmonary fibrosis and cirrhosis. By specifically targeting CAMs, it is possible to disrupt the profibrotic process and potentially attenuate or reverse the fibrosis. The importance of CAMs in cell–cell and cell–matrix interactions and signaling transduction highlights their involvement in various pathogenic conditions, particularly fibrotic disorders. Therefore, a comprehensive understanding of CAMs’ roles in fibrosis is crucial for developing effective therapeutic strategies.
